# Nanoscale Conductive Channels in Silicon Whiskers with Nickel Impurity

**DOI:** 10.1186/s11671-017-1855-9

**Published:** 2017-01-26

**Authors:** Serhii Yatsukhnenko, Anatoly Druzhinin, Igor Ostrovskii, Yuriy Khoverko, Mukhajlo Chernetskiy

**Affiliations:** 1 Lviv Polytechnic National University, 12 S. Bandera Str, 79013 Lviv, Ukraine; 2 International Laboratory of High Magnetic Fields and Low Temperatures, Gajowicka 95, Wroclaw, Poland

**Keywords:** Whiskers, Magnetoresistance, Impurity, Clusters, Polaron

## Abstract

The magnetization and magnetoresistance of Si whiskers doped with <Ni, B> to boron concentrations corresponding to the metal-insulator transition (2 × 10^18^ cm^−3^ ÷ 5 × 10^18^ cm^−3^) were measured at high magnetic fields up to 14 T in a wide temperature range 4.2–300 K. Hysteresis of the magnetic moment was observed for Si p-type whiskers with nickel impurity in a wide temperature range 4.2–300 K indicating a strong interaction between the Ni impurities and the possibility of a magnetic cluster creation. The introduction of Ni impurity in Si whiskers leads to appearance and increase of the magnitude of negative magnetoresistance up to 10% as well as to the decrease of the whisker resistivity in the range of hopping conductance at low temperatures. The abovementioned effects were explained in the framework of appearance of magnetic polarons leading to modification of the conductive channels in the subsurface layers of the whiskers.

## Background

Silicon whiskers have a number of scientific and practical applications [1–6]. For example, they can be used as highly sensitive load cells [7]. The whiskers serve as a basis for strain and pressure sensors which can be used in a wide range of relative strain 10^−5^ ÷ 5 × 10^−3^ r.u. and pressure quantities 1 ÷ 10^4^ kbar [8]. During the recent decades, the whiskers with high strength parameters were investigated. For instance, the limit of strength for Si whiskers with 5 μm in diameter reaches to 5% [9].

On the other hand, a number of size effects in Si whiskers were also established. Particularly, by reducing the diameter of the submicron whiskers to less than 500 nm, one can obtain a decrease of the lattice parameter [10] and a shift of the optical absorption edge towards higher energies [11]. The observed features of Si whiskers are associated with an absence of any defects in the crystal lattice and/or an influence of the whisker surface.

It is well known that pure Si is diamagnetic and has a very weak temperature dependence of the lattice diamagnetic susceptibility [12]. Doping the silicon with impurities with one unpaired electron gives paramagnetic contribution to the general magnetization [13]. Thus, the semi-magnetic materials, which electron spins have no preferential direction before the influence of an external magnetic field, are strongly paramagnetic. Nevertheless, interesting magnetic properties associated with the possible formation of superparamagnetic clusters in whiskers of submicron diameter were observed [13]. Also, a size dependence of the magnetic susceptibility occurs in the whiskers [14], distinguishing them from the bulk samples. The possible reason of such phenomena could be a presence of non-controlling magnetic impurities in the whiskers. Therefore, studies of the magnetic impurity behavior in silicon whiskers are important for understanding the physical processes in the material. It is worthy to note that a sufficient number of such impurities can lead to the formation of magnetic clusters [15–21]. The creation of nanoclusters is promising for design of semiconductor electronics devices expanding their functional parameters and field of applications [22, 23]. At the same time, using information about the spin particle dependence on the whisker structural parameters, one can create the conditions in which magnetic impurities affect the movement of charge carriers in the crystal [24, 25]. Certain results have been provided in our previous paper [26], where an influence of Ni impurity on Si whisker magnetoresistance was investigated. Moreover, sensitive sensor of magnetic field operating in low temperature range was designed. However, the relationship between structural and magneto-transport properties of whiskers was not completely investigated. These new results could be used during the development of new magnetic field sensors, spin valves, etc.

The purpose of the present work is to conduct research of Ni impurity influence on Si whisker magnetoresistance in temperature range 4.2–300 K and high magnetic field up to 14 T.

## Methods

**Fig. 1:**
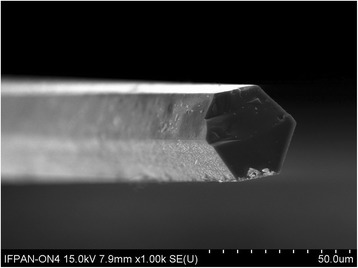
Schematic picture of silicon whisker

As the object of investigation, p-type silicon whiskers were chosen. These whiskers were grown by CVD method in a closed system containing boron, nickel, and gold. Gold was used as growth initiator, while boron and nickel as doping impurities. The temperature of source area was 1370 K and the temperature of crystallization area 1070–1150 K. Si whiskers have transverse size 30–40 μm and length 3–5 mm (Fig. 1). The whiskers were doped with boron during growing process to concentrations correspondent to the vicinity of metal-insulator transition. The critical concentration of metal-insulator transition (MIT) for Si crystal is 5 × 10^18^ cm^−3^ [27]. The calculation of boron concentration was done by Hall method.

We chose nickel for our investigation because it has high solubility and diffusivity in silicon which can facilitate the creation of nanocluster systems [20]. The procedure of doping Si whiskers with nickel consisted of the following stages. At first, nickel film was deposited on the opposite ends of the whisker. Then annealing of the whisker at certain temperatures was fulfilled to diffuse nickel atoms inside the crystal. The diffusion process of nickel into crystal’s volume was conducted at a temperature of 850 °C, which can be considered according to [28] as low temperature diffusion. As a result of the diffusion process, the defined zones of impurities were created, which, on the one hand, provide a formation of the ohmic contacts to the crystal and, on the other hand, have unhomogeneous distribution of impurities from the surface to the crystal volume.

Thus, for the next investigation, two groups of samples were chosen:

1. Group I includes Si whiskers doped with B to concentration of about 5 × 10^18^ cm^−3^ and Si whiskers doped with B to concentration of about 5 × 10^18^ cm^−3^ and additionally doped with Ni.
2. Group II includes Si whiskers with B concentration corresponding to dielectric side of MIT (*N*_B_ = 2 × 10^18^ cm^−3^) with and without nickel, respectively.

It is worthy to note that Ni is not electrically active impurity in silicon [29], but magnetic impurity. Thus, Ni presence in the whiskers should lead to a substantial change in their magnetic and magnetotransport properties.

Faraday method was used for measuring the magnetic susceptibility (MS) of the whiskers in magnetic field within the intensity range 0.3–4.0 kOe at temperatures 4.2–300 K. Before the experiment, the whiskers were packed into cylindrical glass tubes, 3 mm in diameter, and fastened by wax. After that, the samples were pushed out off the glass tube, ready for MS investigation. To account for the wax magnetic properties, a sample with pure wax was also prepared. The experimental results show that the magnetic susceptibility of the wax is much lesser than the MS of the whiskers. Nevertheless, the magnetic contribution of the wax was taken into account in every measurement and used for data correction. It was shown that the measurement error did not exceed 5%.

For magneto-transport investigation, the samples have been cooled down to 4.2 K in the helium cryostat. A special inset with a bifilar winding heater has been used to heat up samples to room temperature. The stabilized electrical current of 1–100 μA depending on the resistance of the sample has been generated by Keithley 224 current source. Digital multimeters Keithley 2000 and Keithley 2010 with simultaneous automatic data registration via parallel port of PC have been used to measure the voltage at potential contacts of the samples, output signals from thermocouple, and magnetic field sensor. The accuracy was up to 1 × 10^−6^ V. The Bitter-magnet based setup has been used to study the effect of strong magnetic fields on the samples. The induction of the magnet was 14 T, deflection time 1.75 and 3.5 T/min at 4.2 K, and higher temperature range, respectively.

## Results

**Fig. 2:**
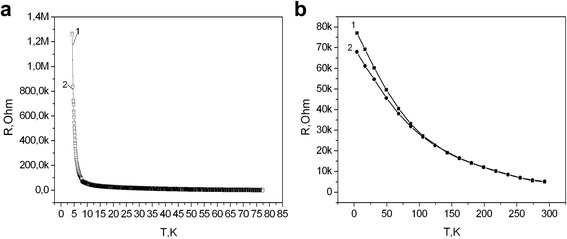
Si <B> whisker resistance versus temperature for the samples doped with boron concentration *N*_B_ = 2 × 10^18^ cm^−3^ which corresponds to dielectric side of the metal-insulator transition (**a**) and boron concentration *N*_B_ = 5 × 10^18^ cm^−3^ which corresponds to the metal-insulator transition (**b**). Curves *1* and *2* corresponds to the samples with and without Ni impurity, respectively

For the investigation of Ni influence on the conductivity mechanisms in Si whiskers, the temperature dependencies of the whisker resistance were measured in temperature range 4.2–300 K. The whiskers with boron concentration corresponding to the dielectric side of MIT (*N*_B_ = 2 × 10^18^ cm^−3^) and the vicinity to MIT (*N*_B_ = 5 × 10^18^ cm^−3^) were under investigation. Figure 2 shows the results of a comparison of temperature behavior of the resistance for the whiskers with (curve 1) and without (curve 2) nickel, respectively.

As you can see from Fig. 2a, the samples with lower B concentration (group II) show the same temperature dependence of the resistance independently on Ni presence or absence. The whiskers with B concentration corresponding to MIT (group I) show Ni influence on temperature dependence of the resistance, especially in the range of low temperatures 4.2–40 K (see Fig. 2b).

As shown in Fig. 2, resistivity of silicon whiskers (Fig. 2b) is subjected to the Mott law at low temperatures (ln*ρ*~T^−1/4^). This confirms the dominance of hopping conductivity at low temperatures [26, 27]:

1 $$ \rho ={\rho}_o\kern0.5em  \exp \kern0.5em {\left({T}_o/ T\right)}^{1/ n}, $$

where *n* = 1/3 at *T* < 10 K; *n* = 1/4 at *T* = 10–25 K; *T*_*o*_ *—*Mott temperature.

Previous studies are consistent with the model which has been proposed by Mott and used in our research. This model explains the hopping conduction of the whiskers at low temperatures. The obtained influence of Ni impurities on the whisker resistance at the region of hopping conductance could be understood taking into account polarization effect of Ni on charge carriers. It is obvious that the effect depends on spin orientation of charge carriers, which is specific at the region of hopping conductance in the sample with B concentration in the vicinity to MIT (group I). A temperature growth leads to an increase of the number of valence charge carriers accompanying spin depolarization as well as to a decrease of Ni impurities influence on the whisker resistance (Fig. 2b). As for the absence of any influence of Ni impurities on the whisker resistance (Fig. 2a), there are no charge carriers with polarized spins in the samples with lower B concentration (group II) in low temperature range. So, Ni impurities are likely influenced on the charge carriers, twice occupied on B impurities, which are present at low temperature range in the sample with concentration in the vicinity to MIT.

**Fig. 3:**
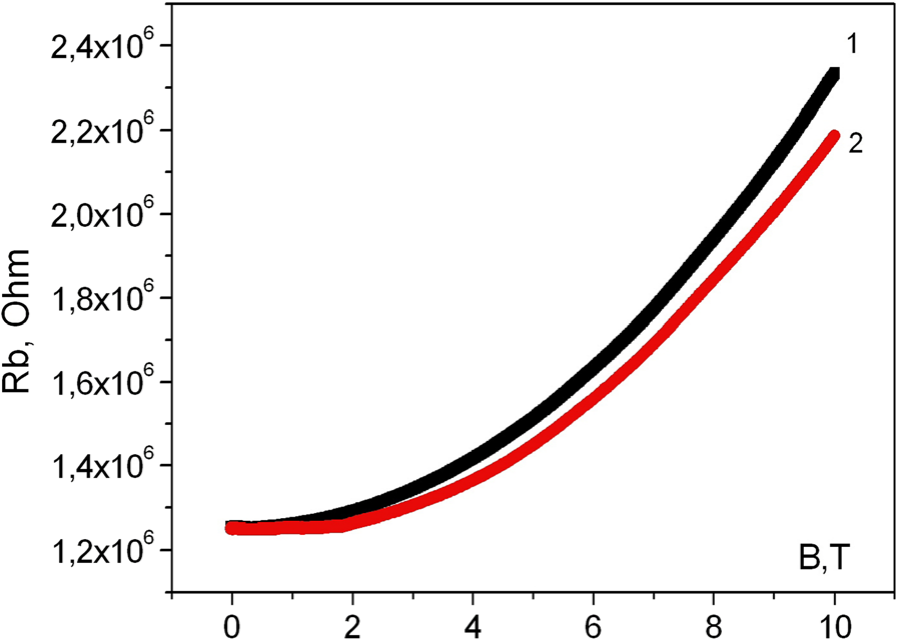
Magnetic field dependence of transverse (*1*) and longitudinal (*2*) magnetoresistance of Si <B, Ni> whiskers with concentration of charge carriers, which corresponds to the dielectric side of MIT (*N*_B_ = 2 × 10^18^ cm^−3^) at *T* = 4.2 K

The further investigations consist measurement of the whisker magnetoresistance at low temperature range. The investigated whiskers with lower B concentration (*N*_B_ = 2 × 10^18^ cm^−3^) have shown significant magnetic field dependence of the resistance, in particular the magnetoresistance increases in 100% at B = 10 T as compared with its magnitude at B = 0 (Fig. 3). As you can see from Fig. 3, longitudinal and transverse magnetoresistance are lightly differed at *T* = 4.2 K, while no change of the whisker magnetoresistance was observed during the Ni impurity presence or absence. So, the difference between the magnetoresistance curves 1 and 2 may be associated with morphological features of the whisker.

**Fig. 4:**
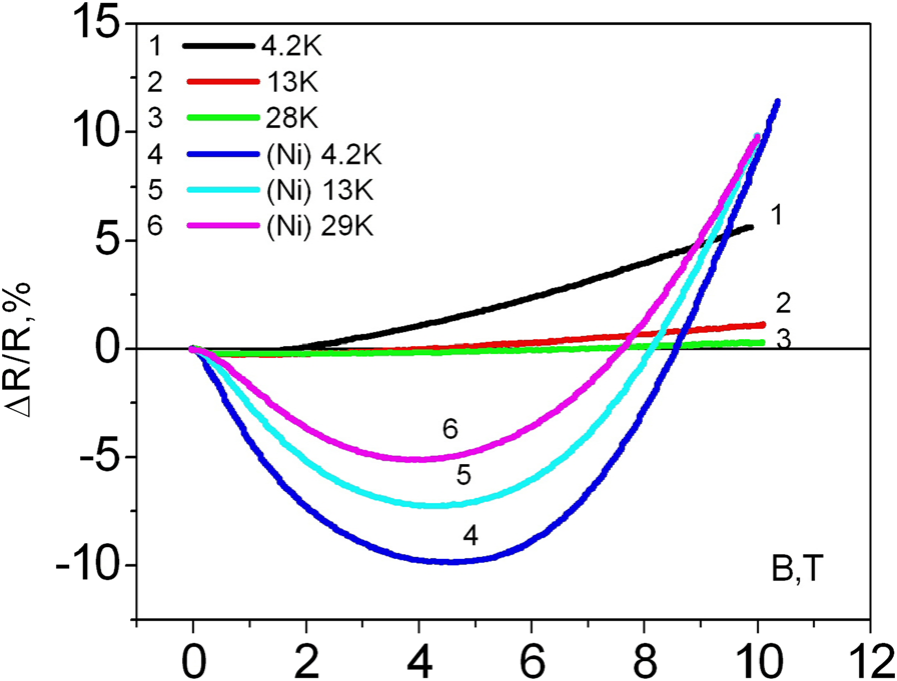
Magnetic field dependencies of resistance of the Si whiskers doped to concentrations that corresponds to MIT (*N*_B_ = 5 × 10^18^ cm^−3^) without Ni impurities (curves *1*–*3*) and with Ni impurity (curves *4*–*6*), respectively

The absence of the manifestation of the negative magnetoresistance (NMR) (Fig. 3) which was observed in [14, 26] can be explained by the low charge carrier concentration. Taking into account the comparison of temperature dependences of resistance (Fig. 2), one can consider the magnetoresistance of the whiskers with higher boron concentration. Figure 4 shows the field dependencies of Si whisker magnetoresistance for samples with boron concentration corresponding to MIT (*N*_B_ = 5 × 10^18^ cm^−3^) without Ni impurities (curves 1–3) and with Ni impurities (curves 4–6), respectively.

The results presented in Fig. 4 show that for all samples with and without Ni, NMR was observed. However, it was found that the impurity of transition metal can significantly expand the temperature range of existence of NMR and facilitate the increase of its values for the concentration corresponding to MIT.

**Fig. 5:**
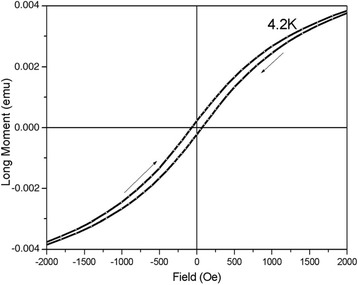
Field dependence of magnetization for silicon whiskers doped with nickel impurity

Several experiments of magnetic susceptibility by the Faraday method were conducted in order to confirm the assumption about the presence of the magnetic moment in the whiskers (Fig. 5).

Hysteresis of the magnetic moment was observed for Si p-type whiskers with nickel impurity in a wide temperature range 4.2–300 K. The hysteresis of magnetic moment exists throughout the entire temperature range. However, the biggest coercive force was obtained at *T* = 4.2 K (Fig. 5), where the biggest negative magnetoresistance occurred.

## Discussion

**Fig. 6:**
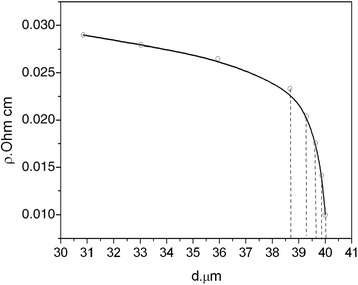
Si whisker resistivity at *T* = 300 K versus its diameter decreased by etching

Our previous works experimentally verified that Si <B> whiskers have unhomogenous distribution of impurities deep into the crystal: impurity concentration decreases from the surface to the center of the crystal. Such conclusion was reached in [12, 27], taking into account the analysis of transverse and longitudinal whisker resistance measurement. In the present paper, we have conducted the direct experiment confirming the above conclusion. The experiment includes a few subsequent stages of etching the surface layers of the crystal and measuring the whisker resistance. The dependence of the whisker resistance on its diameter is shown in Fig. 6. As you can see from Fig. 6, a small etching of the whisker surface (less than 1 μm) leads to substantial increase of the whisker resistance. This fact indicates that the main contribution for the whisker conductance might be attributed to the whisker subsurface layers.

**Fig. 7:**
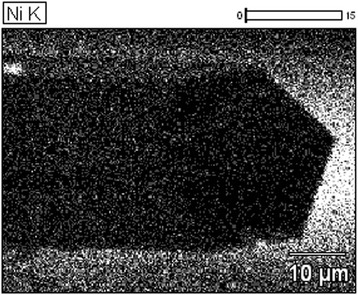
The surface of the silicon whiskers with nickel impurity

Since introduction of Ni impurities in the whiskers was realized from Ni film, it is worthy to investigate the distribution of Ni atoms on the whisker surface. For this purpose, Ni content was investigated with the use of the Camebax technique. The surface profile of Si whiskers is shown in Fig. 7.

As you can see from Fig. 7, Ni atoms are likely joined in clusters on the whisker surface.

**Fig. 8:**
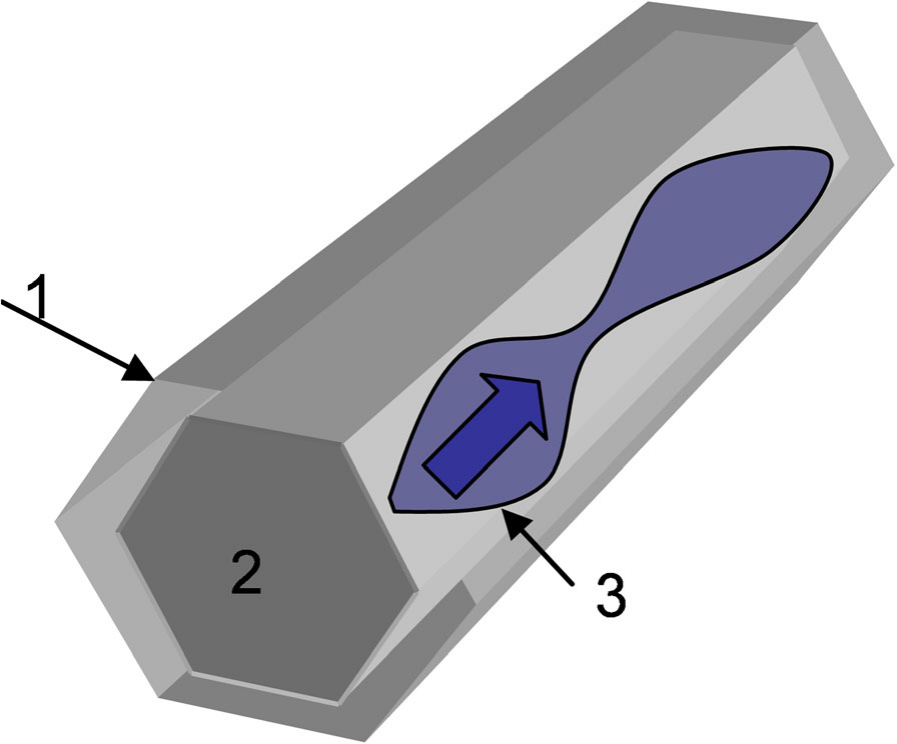
Schematic of magnetic structures formation of the crystal: *1*—surface layer, *2*—core of crystal, and *3*—schematic formation of conducting regions on the surface of the crystal

Nickel is an impurity with high speed diffusion into silicon, and it is obvious that the temperature of diffusion directly affects the depth of the location of magnetic impurities and the formation of the surface layers of the crystal. Localization of impurities can occur both on the surface and in the bulk crystal, depending on the method of alloying, temperature diffusion of impurities, crystal structure, and size of the crystal. Nickel possesses the needed diffusion characteristics for entry into the silicon at low temperature. As was mentioned in the experiment, diffusion was held at the lower limit of solubility, so it was suggested that nickel is located in the subsurface layers of the whiskers (Fig. 8). Taking into account Ni diffusion coefficient 1.34 × 10^−18^ m^2^/c at temperature 850 °C and diffusion time of about 10–15 min, one can calculate the depth of Ni profile in Si—it does not exceed 1–2 μm.

So, taking into account the abovementioned discussion, one can suppose that subsurface layers of the whisker are saturated as B and Ni impurities. Then, the magnetic impurities (Ni) could be polarized by the influence of a magnetic field in the crystals. The polarization of the magnetic impurities, which are located close to each other, could lead to the occurrence of polarized zones, which can affect the movement of charge carriers. By managing such areas, it is possible to create the conductive channels in the crystal, which can be controlled by an external magnetic field.

**Fig. 9:**
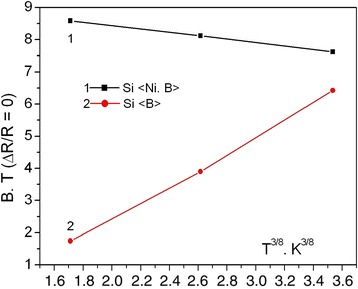
The temperature dependence on the magnetoresistance of silicon microcrystals

It was shown in [26] that for any two temperatures *T*_1_ and *T*_2_, the following equation must be held true:

2 $$ \frac{H_1^0}{H_2^0}\cong {\left(\frac{T_1}{T_2}\right)}^{\frac{1}{2}}, $$

where ${H}_n^0$ is a magnetic field intensity at which the magnetoresistance from negative values becomes equal to zero. Performance of Eq. (2) corresponds to linear change of ${H}_n^0$ as function of temperature ${H}_n\approx {T}^{\frac{3}{8}}$. Taking into account the results of Fig. 4, one can draw the corresponding dependencies for Si whiskers with and without nickel impurity (see Fig. 9).

As shown in Fig. 9, the observed dependencies substantially differ. You can see typical ${H}_n\approx {T}^{\frac{3}{8}}$ dependency for the whiskers doped with B impurities. The dependency corresponds to the presence of Mott conductance in the samples at low temperatures. However, for the whiskers doped with B and Ni impurities, the incline of dependency is significantly different from the previous curve. A possible explanation for the observed differences is the influence of the magnetic impurities on the crystal parameters, in particular creation of magnetic polarons [30].

Taking into account the whisker magnetization (Fig. 5), it is obvious that in the subsurface region of the whisker, there is some magnetic ordering influenced on magnetotransport properties. These clusters are characterized by the orientation in the magnetic field as well as the number of elements in the cell. With enough quantity of a magnetic impurity, the clusters will cause the polarization effects around them—will act as a polaron.

Obviously, the impact of magnetic clusters on the magnetotransport properties of the whiskers should be significant at low temperatures. The polarization of magnetic clusters and its nearest neighbors occurs after lowering the temperature, which directly affects the process of charge carriers’ movement [30]. If the distance between clusters is enough for interaction, a conductive channel that accelerates the movement of charge can be formed. That is, when the charge carrier enters in the interaction field of one cluster, the acceleration of them to another cluster occurs. As a result, one can see an abrupt decrease of the whisker resistivity (Fig. 2b) as well as occurrence of NMR in low magnetic fields (Fig. 4).

Having the possibility to control the charge movement, one can use Si whiskers as a switch, spin valve, etc. This allows the use of silicon whiskers in spintronics.

## Conclusions

In the present work, the investigation of Si whiskers doped with <Ni, B> to boron concentrations correspondent to MIT (2 × 10^18^ cm^−3^ ÷ 5 × 10^18^ cm^−3^) was conducted at high magnetic fields up to 14 T in a wide range of temperatures 4.2–300 K. The influence of Ni content on the whisker resistance and magnetoresistance was studied. In particular, a large change of negative magnetoresistance was found in the whiskers containing magnetic impurities. The investigations have shown that the nickel impurity enhances the manifestation of negative magnetoresistance effect to 10%. Besides, Ni impurities localized in the subsurface region of Si whiskers were found to decrease a magnitude of the whisker resistance in the range of hopping conductance at low temperatures.

To explain the above effect, we have used additional experiment consisting in subsequent etching the surface layers of the crystal and measuring the whisker resistance. The etching of the whisker surface leads to substantial increase of the whisker resistance indicating that the whisker subsurface layers are the main contributors for the whisker conductance.

The investigation of the whisker magnetization, i.e., existence of hysteresis of magnetization in wide range of temperatures 4.2–300 K, has shown that magnetic ordering takes place in the whiskers. Taking into account small diffusion depth of Ni atoms (of about 1–2 μm), one can suppose that magnetic clusters of Ni impurities are likely created in the whiskers.

An analysis of NMR in the whisker doped with B and Ni has shown a probable creation of magnetic polarons in the subsurface layers of the whisker. The polarons may affect the transport of charge carriers, in particular lead to occurrence of conductive channels for charge carriers which could be controlled by applying magnetic field. Having the possibility to control the charge carrier movement, one can use Si whiskers in spintronics devices.

## Abbreviations

CVD: Chemical vapor deposition
MIT: Metal-insulator transition
MS: Magnetic susceptibility
NMR: Negative magnetoresistance
